# Supplementary material for: Modeling glioblastoma heterogeneity as a dynamic network of cell states
Source: Mol Syst Biol. 2021 Sep 16;17(9):e10105. doi: 10.15252/msb.202010105 (PMC8444284; doi:10.15252/msb.202010105)
Supplement: Supplementary file 6 — Source Data for Figure 5 [file MSB-17-e10105-s004.zip › Figure5A_sourcedata/GSEA_3017/nosigdb_stateC.GseaPreranked.1622795002785/RICHARDS_NOWAKOWSKI_SCIENCE_2017_ORG_UPREG.html]

Details for gene set RICHARDS\_NOWAKOWSKI\_SCIENCE\_2017\_ORG\_UPREG[GSEA]

|  || Dataset | state13017 |
| Phenotype | NoPhenotypeAvailable |
| Upregulated in class | na\_neg |
| GeneSet | RICHARDS\_NOWAKOWSKI\_SCIENCE\_2017\_ORG\_UPREG |
| Enrichment Score (ES) | -0.38716438 |
| Normalized Enrichment Score (NES) | -2.9222713 |
| Nominal p-value | 0.0 |
| FDR q-value | 0.0 |
| FWER p-Value | 0.0 |
Table: GSEA Results Summary

  

Fig 1: Enrichment plot: RICHARDS\_NOWAKOWSKI\_SCIENCE\_2017\_ORG\_UPREG      
 Profile of the Running ES Score & Positions of GeneSet Members on the Rank Ordered List

  

| PROBE | GENE SYMBOL | GENE\_TITLE | RANK IN GENE LIST | RANK METRIC SCORE | RUNNING ES | CORE ENRICHMENT || 1 | MAGI1 |  |  | 6 | 0.652 | -0.0029 | No |
| 2 | HIST1H1C |  |  | 11 | 0.583 | -0.0025 | No |
| 3 | FAM111A |  |  | 13 | 0.576 | 0.0047 | No |
| 4 | ZBTB20 |  |  | 30 | 0.482 | -0.0237 | No |
| 5 | CDCA7L |  |  | 37 | 0.453 | -0.0299 | No |
| 6 | ETV1 |  |  | 44 | 0.438 | -0.0364 | No |
| 7 | PHIP |  |  | 61 | 0.387 | -0.0663 | No |
| 8 | GINS2 |  |  | 83 | 0.360 | -0.1081 | No |
| 9 | BARD1 |  |  | 86 | 0.357 | -0.1068 | No |
| 10 | CARHSP1 |  |  | 90 | 0.353 | -0.1078 | No |
| 11 | CKB |  |  | 102 | 0.335 | -0.1273 | No |
| 12 | STAG2 |  |  | 128 | 0.309 | -0.1789 | No |
| 13 | SSX2IP |  |  | 131 | 0.307 | -0.1784 | No |
| 14 | SLC12A2 |  |  | 148 | 0.288 | -0.2100 | No |
| 15 | PARP1 |  |  | 163 | 0.280 | -0.2372 | No |
| 16 | SNCAIP |  |  | 182 | 0.264 | -0.2736 | No |
| 17 | NAP1L4 |  |  | 184 | 0.262 | -0.2716 | No |
| 18 | E2F3 |  |  | 194 | 0.252 | -0.2879 | No |
| 19 | KAZN |  |  | 200 | -0.252 | -0.2951 | No |
| 20 | CAST |  |  | 205 | -0.253 | -0.3001 | No |
| 21 | SHC3 |  |  | 206 | -0.253 | -0.2959 | No |
| 22 | ARHGAP12 |  |  | 207 | -0.254 | -0.2918 | No |
| 23 | HEPACAM |  |  | 216 | -0.257 | -0.3057 | No |
| 24 | PGAM1 |  |  | 218 | -0.258 | -0.3038 | No |
| 25 | FERMT2 |  |  | 227 | -0.259 | -0.3177 | No |
| 26 | MBNL2 |  |  | 233 | -0.260 | -0.3247 | No |
| 27 | ZYX |  |  | 236 | -0.261 | -0.3250 | No |
| 28 | CLIC1 |  |  | 237 | -0.261 | -0.3208 | No |
| 29 | CCDC6 |  |  | 240 | -0.262 | -0.3210 | No |
| 30 | SAT1 |  |  | 241 | -0.262 | -0.3167 | No |
| 31 | FDFT1 |  |  | 243 | -0.262 | -0.3147 | No |
| 32 | ZFYVE21 |  |  | 247 | -0.263 | -0.3172 | No |
| 33 | PCGF5 |  |  | 252 | -0.264 | -0.3220 | No |
| 34 | NPAS3 |  |  | 256 | -0.265 | -0.3244 | No |
| 35 | ID4 |  |  | 261 | -0.267 | -0.3291 | No |
| 36 | PPIB |  |  | 265 | -0.268 | -0.3315 | No |
| 37 | ANAPC16 |  |  | 268 | -0.269 | -0.3317 | No |
| 38 | GATM |  |  | 272 | -0.270 | -0.3341 | No |
| 39 | HSD17B12 |  |  | 277 | -0.271 | -0.3387 | No |
| 40 | HEATR5A |  |  | 278 | -0.271 | -0.3343 | No |
| 41 | DTNA |  |  | 281 | -0.271 | -0.3344 | No |
| 42 | SQLE |  |  | 293 | -0.274 | -0.3549 | No |
| 43 | PHLDA1 |  |  | 295 | -0.274 | -0.3527 | No |
| 44 | L3HYPDH |  |  | 298 | -0.276 | -0.3527 | No |
| 45 | SPAG16 |  |  | 300 | -0.277 | -0.3504 | No |
| 46 | HERPUD1 |  |  | 302 | -0.277 | -0.3482 | No |
| 47 | CNKSR3 |  |  | 305 | -0.280 | -0.3481 | No |
| 48 | NAMPT |  |  | 306 | -0.280 | -0.3435 | No |
| 49 | SLC1A3 |  |  | 307 | -0.281 | -0.3390 | No |
| 50 | CREB5 |  |  | 309 | -0.281 | -0.3366 | No |
| 51 | LIFR |  |  | 311 | -0.282 | -0.3343 | No |
| 52 | NUDT4 |  |  | 321 | -0.285 | -0.3500 | No |
| 53 | DHCR7 |  |  | 322 | -0.285 | -0.3454 | No |
| 54 | HS6ST1 |  |  | 328 | -0.287 | -0.3520 | No |
| 55 | CRISPLD1 |  |  | 331 | -0.288 | -0.3518 | No |
| 56 | MVD |  |  | 333 | -0.288 | -0.3494 | No |
| 57 | TUBB2A |  |  | 336 | -0.289 | -0.3492 | No |
| 58 | SHISA5 |  |  | 349 | -0.297 | -0.3716 | No |
| 59 | MEST |  |  | 352 | -0.299 | -0.3712 | No |
| 60 | ITGB5 |  |  | 356 | -0.300 | -0.3731 | No |
| 61 | UGDH |  |  | 360 | -0.301 | -0.3750 | No |
| 62 | LRP1 |  |  | 366 | -0.304 | -0.3813 | No |
| 63 | SPRY1 |  |  | 369 | -0.307 | -0.3809 | No |
| 64 | PDPN |  |  | 372 | -0.307 | -0.3804 | No |
| 65 | LMNA |  |  | 376 | -0.308 | -0.3821 | Yes |
| 66 | FOS |  |  | 377 | -0.309 | -0.3771 | Yes |
| 67 | CSPG5 |  |  | 381 | -0.310 | -0.3788 | Yes |
| 68 | IGFBP5 |  |  | 384 | -0.311 | -0.3783 | Yes |
| 69 | SAMD4A |  |  | 388 | -0.313 | -0.3799 | Yes |
| 70 | CHST3 |  |  | 389 | -0.314 | -0.3748 | Yes |
| 71 | CETN2 |  |  | 392 | -0.317 | -0.3742 | Yes |
| 72 | S100A13 |  |  | 396 | -0.319 | -0.3758 | Yes |
| 73 | PLTP |  |  | 397 | -0.319 | -0.3706 | Yes |
| 74 | TIMP1 |  |  | 399 | -0.320 | -0.3676 | Yes |
| 75 | ZFP36L2 |  |  | 401 | -0.321 | -0.3646 | Yes |
| 76 | CDH4 |  |  | 402 | -0.321 | -0.3594 | Yes |
| 77 | FGFR3 |  |  | 403 | -0.322 | -0.3541 | Yes |
| 78 | SIPA1L1 |  |  | 412 | -0.329 | -0.3669 | Yes |
| 79 | SLC4A4 |  |  | 415 | -0.332 | -0.3660 | Yes |
| 80 | HMGCR |  |  | 418 | -0.334 | -0.3650 | Yes |
| 81 | LDLR |  |  | 419 | -0.334 | -0.3596 | Yes |
| 82 | CALD1 |  |  | 420 | -0.335 | -0.3541 | Yes |
| 83 | SLC2A3 |  |  | 422 | -0.336 | -0.3509 | Yes |
| 84 | PMP2 |  |  | 424 | -0.337 | -0.3476 | Yes |
| 85 | RHOQ |  |  | 426 | -0.338 | -0.3444 | Yes |
| 86 | KLF6 |  |  | 429 | -0.339 | -0.3434 | Yes |
| 87 | REXO2 |  |  | 432 | -0.342 | -0.3423 | Yes |
| 88 | INSIG1 |  |  | 434 | -0.342 | -0.3390 | Yes |
| 89 | VEGFA |  |  | 436 | -0.345 | -0.3356 | Yes |
| 90 | WWTR1 |  |  | 437 | -0.345 | -0.3300 | Yes |
| 91 | FAT1 |  |  | 441 | -0.346 | -0.3311 | Yes |
| 92 | RAB31 |  |  | 445 | -0.349 | -0.3322 | Yes |
| 93 | HSPA5 |  |  | 447 | -0.350 | -0.3288 | Yes |
| 94 | MYL12A |  |  | 448 | -0.350 | -0.3231 | Yes |
| 95 | BCAN |  |  | 449 | -0.351 | -0.3173 | Yes |
| 96 | IDI1 |  |  | 451 | -0.353 | -0.3138 | Yes |
| 97 | NEK6 |  |  | 453 | -0.355 | -0.3103 | Yes |
| 98 | DKK3 |  |  | 454 | -0.356 | -0.3045 | Yes |
| 99 | RHOC |  |  | 455 | -0.359 | -0.2986 | Yes |
| 100 | SEPT9 |  |  | 457 | -0.361 | -0.2950 | Yes |
| 101 | SLITRK2 |  |  | 458 | -0.361 | -0.2891 | Yes |
| 102 | EDNRB |  |  | 462 | -0.367 | -0.2899 | Yes |
| 103 | SERPINB6 |  |  | 463 | -0.367 | -0.2839 | Yes |
| 104 | B2M |  |  | 468 | -0.371 | -0.2869 | Yes |
| 105 | HES1 |  |  | 470 | -0.373 | -0.2830 | Yes |
| 106 | LGALS3 |  |  | 471 | -0.374 | -0.2769 | Yes |
| 107 | CYP51A1 |  |  | 476 | -0.378 | -0.2798 | Yes |
| 108 | HBEGF |  |  | 480 | -0.386 | -0.2803 | Yes |
| 109 | VCL |  |  | 484 | -0.388 | -0.2808 | Yes |
| 110 | ARL6IP5 |  |  | 490 | -0.396 | -0.2856 | Yes |
| 111 | C1orf61 |  |  | 491 | -0.396 | -0.2791 | Yes |
| 112 | FABP7 |  |  | 495 | -0.411 | -0.2792 | Yes |
| 113 | PLIN3 |  |  | 496 | -0.411 | -0.2725 | Yes |
| 114 | LDHA |  |  | 498 | -0.412 | -0.2680 | Yes |
| 115 | JUN |  |  | 503 | -0.425 | -0.2702 | Yes |
| 116 | SCD |  |  | 504 | -0.431 | -0.2631 | Yes |
| 117 | F3 |  |  | 505 | -0.432 | -0.2560 | Yes |
| 118 | SLC25A18 |  |  | 507 | -0.433 | -0.2512 | Yes |
| 119 | SCRG1 |  |  | 508 | -0.434 | -0.2441 | Yes |
| 120 | NES |  |  | 509 | -0.435 | -0.2370 | Yes |
| 121 | DBI |  |  | 514 | -0.440 | -0.2389 | Yes |
| 122 | CD9 |  |  | 515 | -0.443 | -0.2316 | Yes |
| 123 | PTN |  |  | 517 | -0.446 | -0.2266 | Yes |
| 124 | CA2 |  |  | 518 | -0.448 | -0.2193 | Yes |
| 125 | AHNAK |  |  | 519 | -0.449 | -0.2120 | Yes |
| 126 | CST3 |  |  | 520 | -0.452 | -0.2046 | Yes |
| 127 | MSMO1 |  |  | 525 | -0.460 | -0.2061 | Yes |
| 128 | HMGCS1 |  |  | 529 | -0.466 | -0.2053 | Yes |
| 129 | IFITM3 |  |  | 533 | -0.477 | -0.2043 | Yes |
| 130 | COL6A1 |  |  | 534 | -0.479 | -0.1965 | Yes |
| 131 | ITGA6 |  |  | 535 | -0.479 | -0.1886 | Yes |
| 132 | TTYH1 |  |  | 540 | -0.496 | -0.1896 | Yes |
| 133 | LRRN3 |  |  | 543 | -0.504 | -0.1859 | Yes |
| 134 | HOPX |  |  | 546 | -0.513 | -0.1820 | Yes |
| 135 | GPC1 |  |  | 555 | -0.536 | -0.1914 | Yes |
| 136 | SPARCL1 |  |  | 557 | -0.544 | -0.1848 | Yes |
| 137 | S100B |  |  | 561 | -0.561 | -0.1824 | Yes |
| 138 | CREM |  |  | 562 | -0.563 | -0.1732 | Yes |
| 139 | ANXA2 |  |  | 563 | -0.567 | -0.1640 | Yes |
| 140 | TNC |  |  | 564 | -0.572 | -0.1546 | Yes |
| 141 | MT2A |  |  | 566 | -0.575 | -0.1475 | Yes |
| 142 | EMP3 |  |  | 568 | -0.580 | -0.1403 | Yes |
| 143 | S100A16 |  |  | 569 | -0.582 | -0.1307 | Yes |
| 144 | ITPR2 |  |  | 572 | -0.634 | -0.1249 | Yes |
| 145 | FGFBP3 |  |  | 573 | -0.644 | -0.1144 | Yes |
| 146 | GFAP |  |  | 574 | -0.659 | -0.1036 | Yes |
| 147 | CD44 |  |  | 575 | -0.659 | -0.0928 | Yes |
| 148 | VIM |  |  | 578 | -0.674 | -0.0863 | Yes |
| 149 | NEAT1 |  |  | 579 | -0.683 | -0.0752 | Yes |
| 150 | CLU |  |  | 581 | -0.690 | -0.0662 | Yes |
| 151 | CYR61 |  |  | 584 | -0.714 | -0.0590 | Yes |
| 152 | ATP1B2 |  |  | 585 | -0.714 | -0.0473 | Yes |
| 153 | CA12 |  |  | 586 | -0.717 | -0.0356 | Yes |
| 154 | PMP22 |  |  | 590 | -0.837 | -0.0288 | Yes |
| 155 | MT3 |  |  | 591 | -0.837 | -0.0151 | Yes |
| 156 | FABP5 |  |  | 596 | -1.477 | -0.0000 | Yes |
Table: GSEA details [plain text format]

  

Fig 2: RICHARDS\_NOWAKOWSKI\_SCIENCE\_2017\_ORG\_UPREG: Random ES distribution      
 Gene set null distribution of ES for **RICHARDS\_NOWAKOWSKI\_SCIENCE\_2017\_ORG\_UPREG**
